# How mobile are protons in the structure of dental glass ionomer cements?

**DOI:** 10.1038/srep08972

**Published:** 2015-03-10

**Authors:** Ana R. Benetti, Johan Jacobsen, Benedict Lehnhoff, Niels C. R. Momsen, Denis V. Okhrimenko, Mark T. F. Telling, Nikolay Kardjilov, Markus Strobl, Tilo Seydel, Ingo Manke, Heloisa N. Bordallo

**Affiliations:** 1 Department of Odontology, Faculty of Health and Medical Sciences, University of Copenhagen, DK-2200, Copenhagen, Denmark; 2 The Niels Bohr Institute, University of Copenhagen, DK-2100, Copenhagen, Denmark; 3 European Spallation Source ESS AB, PO Box 176, SE-221 00 Lund, Sweden; 4 Nano-Science Center, Department of Chemistry, University of Copenhagen, DK-2100, Copenhagen, Denmark; 5 ISIS Facility, Rutherford Appleton Laboratory, Chilton, Oxon, UK OX11 0QX; 6 Department of Materials, University of Oxford, Parks Road, Oxford, UK; 7 Helmholtz Zentrum Berlin, D-14109, Berlin, Germany; 8 Institut Laue-Langevin, BP 156, F-38042, Grenoble, France

## Abstract

The development of dental materials with improved properties and increased longevity can save costs and minimize discomfort for patients. Due to their good biocompatibility, glass ionomer cements are an interesting restorative option. However, these cements have limited mechanical strength to survive in the challenging oral environment. Therefore, a better understanding of the structure and hydration process of these cements can bring the necessary understanding to further developments. Neutrons and X-rays have been used to investigate the highly complex pore structure, as well as to assess the hydrogen mobility within these cements. Our findings suggest that the lower mechanical strength in glass ionomer cements results not only from the presence of pores, but also from the increased hydrogen mobility within the material. The relationship between microstructure, hydrogen mobility and strength brings insights into the material's durability, also demonstrating the need and opening the possibility for further research in these dental cements.

The placement of fillings is one the most frequent type of dental treatment. However, a number of these restorations fail within a short period of time^1^,^2^,^3^,^4^. As a result, general practitioners spend a considerable part of their time replacing failed restorations. After caries, fracture of the material is among the main reasons for replacement^4^,^5^,^6^. Moreover, the larger the restoration the greater the rate of replacement, particularly in high-load-bearing areas^1^,^3^,^4^,^7^. Therefore, the worldwide costs associated with the provision of dental restorations are worth billions of dollars for individuals and the society. For instance, the National Health Service in England and Wales has reported an annual expense of approximately £320 million in dental restorative work^8^. In Denmark, the public expense on oral care reached €455 million in 2005^9^. These numbers point out the need for advances in dental treatments, focusing on preventive therapies and the development of improved restorative materials^10^. In particular, the development of high-quality, mercury-free materials for dental restorations has become a higher priority following the Minamata Convention on Mercury in 2013^11^. This treaty signed by 140 countries seeks to minimize the effects of mercury on the environment by reducing mercury emissions worldwide from all sources, including dental practices. According to this agreement, the use of dental amalgam shall be phased down, posing the need for the development of new mercury-free restorative materials^10^,^11^, with enhanced longevity in the challenging oral environment.

Among the mercury-free alternatives, tooth-colored resin composites and glass ionomer cements (GIC) are the available restorative options. Resin composites are mechanically stronger and show increased longevity when compared to GIC^1^,^4^,^10^. Despite the lower mechanical strength of GIC, however, these materials have several desirable properties for dental restorations, such as: 1) thermal expansion coefficients similar to the hard dental tissues; 2) chemical bonding to enamel and dentine, without the need of an intermediate bonding agent; 3) release of fluoride, with consequent positive effects on the re- and de-mineralization processes involved in the formation of carious lesions; 4) biocompatibility and 5) antimicrobial properties^12^,^13^. These properties make GIC suitable for a minimally invasive care approach in the promotion of global oral health. Additionally, GIC are indicated for the atraumatic restorative treatment, also known as the ART technique^14^. This technique, in which carious lesions are filled with a high-viscosity GIC after excavation using hand instruments, has shown good clinical results in populations that do not have access to more elaborate dental treatments^14^,^15^,^16^. In order to expand the clinical use and durability of GIC and further benefit from their favourable characteristics, an improvement in their mechanical properties is paramount^13^. This can be achieved, for example, by a better understanding of the chemical reactions and hydration that occur during setting^17^, which may allow designing a better controlled process.

GIC set by neutralization after the mixing of aluminosilicate glass particles, containing phosphate and fluoride, with a polyacid solution. From previous studies^18^,^19^,^20^,^21^,^22^,^23^, two steps in this diffusion-controlled reaction have been identified. During the earlier stages of setting, the acid partially dissolves the glass particles resulting in the release of metal ions^24^,^25^, for example calcium, strontium, and aluminium^25^. These metal ions crosslink with the polymeric acid molecules, thus triggering the formation of carboxylate salts^23^,^26^. Calcium or strontium carboxylates are formed promptly within the first minute after the mixture^26^. Subsequently, crosslinking of the aluminium ions with the acidic polymer chains results in the formation of aluminium carboxylate salts^12^,^27^,^28^,^29^,^30^, a process identified to start after approximately ten minutes^26^ and to be completed mostly within the first day^27^,^29^.

Indeed previous studies using acid-washing, Fourier-transform infrared (FTIR), Raman as well as nuclear magnetic resonance (NMR) spectroscopies have brought a comprehensive understanding of the chemical reactions involved in the setting of GIC. The continued crosslinking between the metal ions and the polyacid was originally considered responsible for further hardening of the cement, with consequent improvement in strength^12^,^13^,^20^. Later work showed evidence that, in addition to the formation of crosslinks with the polyacid, the leached ions are also involved in the formation of a hydrated inorganic network, considered much important to the improvement in strength of these cements^17^,^20^,^21^. However, the nature of the hydration processes involved in the setting is yet not fully understood. For instance, although earlier studies hypothesised that the inorganic network is a silicate^17^,^20^,^21^, more recent work suggests that it is phosphate-based^31^,^32^,^33^. Nevertheless, it is agreed that the ratio of bound to unbound water increases with ageing of the cement samples^17^,^34^. Undoubtedly, water plays an important role in the setting reaction and in the formation of the hydrated inorganic network. It is also known that whilst hardening of GIC occurs within a few minutes, slower reactions involved in the maturation of these cements last several months^20^. Therefore, a better understanding of the hydration processes in relation to the development of the pore structure and strength are required.

The knowledge about hydration in GIC can be extended by using neutron spectroscopy, a technique able to disentangle the process of crosslinking from the formation of the hydrated inorganic network, simply because these processes occur in different time-scales. While the faster water dynamics above −23°C (250 K) are easily accessible on the pico-second (ps) time-scale, the acceleration of the slower polymeric motions due to thermal activation can be visible in the nano-second (ns) time-scale. In particular, the use of quasi-elastic neutron scattering (QENS) spectroscopy gives a unique perception of the hydrogen motions within cement pastes^34^,^35^,^36^. When neutrons interact with the sample, scattering occurs and produces a peak broadening of the initial energy distribution. Analysis of the angular and energy distribution allows the dynamics and associated geometric motion of the hydrogen atoms to be investigated from ps to ns time-scales^37^. By analysing the area under the peak, in the defined interval of the initial energy distribution (hereon called elastic intensity), information about the number of immobile protons can be obtained. This is the so-called elastic fixed window (EFW) approach^35^. The evolution of the number of immobile protons during hydration can therefore be followed in terms of an immobile hydrogen index (IHI, see supplementary information online for details).

While neutron spectroscopy brings information about the mobility of hydrogen in the cement structure, it is limited in providing insight into the development of the structural architecture. To this end, the microstructure of GIC has traditionally been assessed via stereomicroscopy or scanning electron microscopy^38^,^39^,^40^. However, these techniques are limited to only two dimensions. Additionally, the specimen preparation required for such techniques disturbs the system in order to expose and visualize the pores. Non-destructive three-dimensional investigations of the GIC microstructure using X-rays are rare^41^, but when complemented by other methods^42^,^43^, can bring insight into the relationship between porosity and mechanical strength^40^,^41^,^42^,^43^. Despite the fact that the mechanical strength of GIC improves with ageing^44^,^45^,^46^, most studies do not follow the evolution of the GIC microstructure with time in a systematic manner^38^,^39^,^41^,^42^,^43^. Therefore, to the best knowledge of the authors, accurate information regarding the development of the pore structure following the slow but progressive setting reaction is still missing. A better understanding on the formation of the complex porous structure and the liquid mobility within this structure might contribute to identifying where improvements are possible. One of the most promising techniques to assess the pore structure without disturbances in the sample is neutron imaging, a technique successfully applied to materials science^47^ and the study of construction cements^48^. Neutrons, similarly to X-rays, penetrate matter. However, unlike X-rays, neutrons interact with matter in a different manner, thus allowing the identification of elements with very low molecular weight, including hydrogen^47^. While X-rays allow the characterization of the microstructure of materials^41^, neutron imaging provides information on proton distribution within the structure^35^,^48^,^49^. For this reason, both X-rays and neutron imaging, complemented by biaxial flexural strength (BFS), were applied to achieve a better understanding of the relationship between structure and strength of GIC during the development of the setting reaction. From the ensemble of our results, a better understanding of microstructure and the proton dynamics of the dental glass ionomer cements was achieved.

## Results

Both investigated GIC cements consisted of a powder containing irregular particles of fluoroaluminosilicate glass that undergo, as described previously, an acid-base reaction on contact with the liquid. In the Poly cement (Ionofil Molar AC, Voco GmbH, Germany), the liquid contains a polyacid solution, while in the Aqua (Aqua Ionofil Plus, Voco GmbH, Germany), as the polyacid is freeze-dried and incorporated into the powder, the liquid is water.

**Figure 1:**
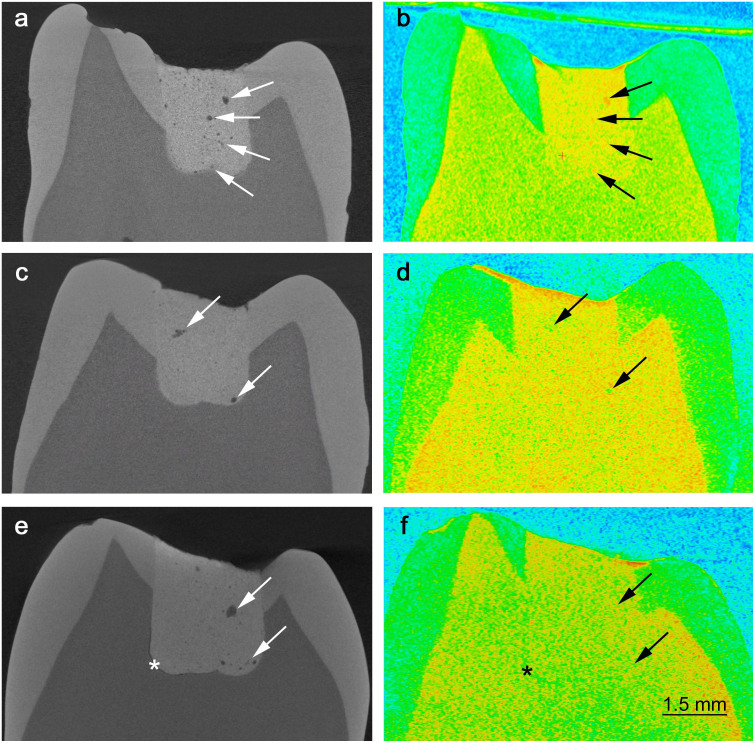
Extracted teeth restored with GIC. Pores and cracks are better visible in the X-ray images due to better resolution (a, c, e). Poor adaptation (*) of the more viscous restorative cement (Poly) at the bottom of the cavity is observed (e), while this problem is less evident for the less viscous cement (a, c). The presence of liquid inside or adhered to the internal walls of some of the larger pores is evident (in red, due to the higher attenuation coefficient of hydrogen) in the neutron image (b). The neutron images also suggest that interconnecting pores or cracks are filled with liquid (b, f), while some of the larger pores seem to be empty (d).

**Figure 2:**
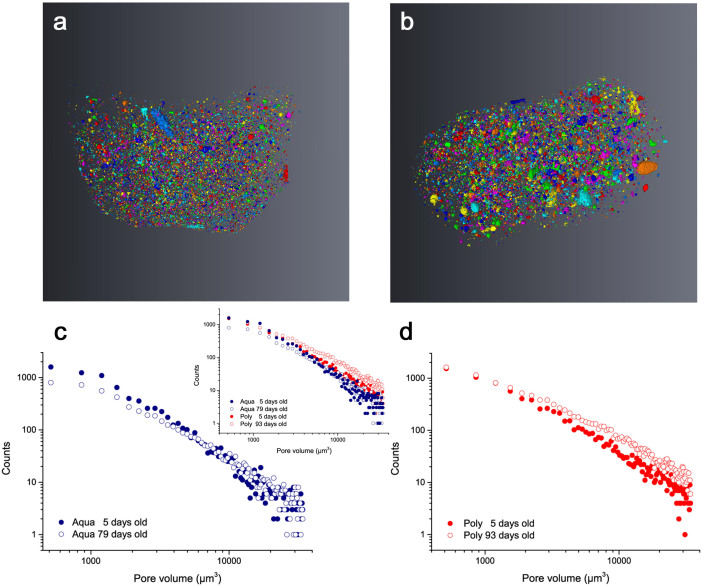
The complex 3D pore structure of GIC is visible in the reconstructed and binarised X-ray images. Smaller pores are visible for the less viscous cement, Aqua (a). This is confirmed by the counts of pores (y axis) according to their volume (x axis), here plotted in a log-log scale. With ageing, a decrease in the count of smaller pores for Aqua is observed, thus indicating that the pore volume becomes smaller than the resolution of the instrument (c). Larger pores are visible in the more viscous cement, Poly (b). With ageing, an overall reduction in the pore size was also observed for the latter, although no significant changes in the small pore range can be noticed (d).

Better resolution provided by X-ray imaging, in contrast to neutron images, allowed clear observation of the microstructure of the GIC restorations. The reconstructed images confirmed the presence of voids of various sizes and shapes within the restorative materials (Figures 1a, 1c, 1e). Isolated or interconnected larger pores were observed. Features such as the presence of cracks and poor adaptation of the restorations were also observed (Figures 1a, 1c, 1e). The less viscous material (Aqua) adapted better to the dental cavity, whereas areas of poor contact between the cement and the tooth were identified in the restoration made with the more viscous cement (Poly, Figure 1e). The pore analysis performed on stacked reconstructed μCT tomography volumetric data confirmed that pore volume distribution in the GIC is complex and highly variable (Figures 2a, 2b). A similar slope was observed for the pore distribution of both investigated GIC. However, differences in the count and volume of the pores were identified (Figures 2c, 2d). From the measured pore volumes, an estimate of the pore diameter was calculated to vary between circa 7 μm and 30 μm, assuming that the pores were approximately spherical. In the aged samples, a reduction of the pore size was observed for both materials (Figures 2c, 2d). In addition, a smaller number of larger pores were observed in the less viscous GIC after 5 days (Figures 2c, 2d).

### Neutron imaging reveals the concentration of hydrogen within the material structure

The analysis of the neutron images suggests the presence of unreacted liquid either inside (Figure 1b), or adsorbed to the internal walls (Figures 1d, 1f), in a number of the larger pores. However, the presence of liquid could neither be identified inside smaller pores nor in some of the larger pores (Figures 1d, 1f ).

### Quasi-elastic neutron scattering demonstrates proton mobility within the dental cements as a function of time and temperature

As the cement paste hydrates, the viscosity of the paste increases together with the degree of structural order. During the maturation process and subsequent evolution of the pore structure, the establishment of hydrogen bonds was probed as a function of time. In order to access the evolution of the microstructure of GIC during the hydration process, the elastic intensity from the QENS measurements was followed for the first 24 h and assessed again at the 5^th^ day. As the material sets, more hydrogen bonds are formed and thus the amount of immobile hydrogen increases. Therefore, the immobile hydrogen index (IHI) can be directly related to the cement setting.

**Figure 3:**
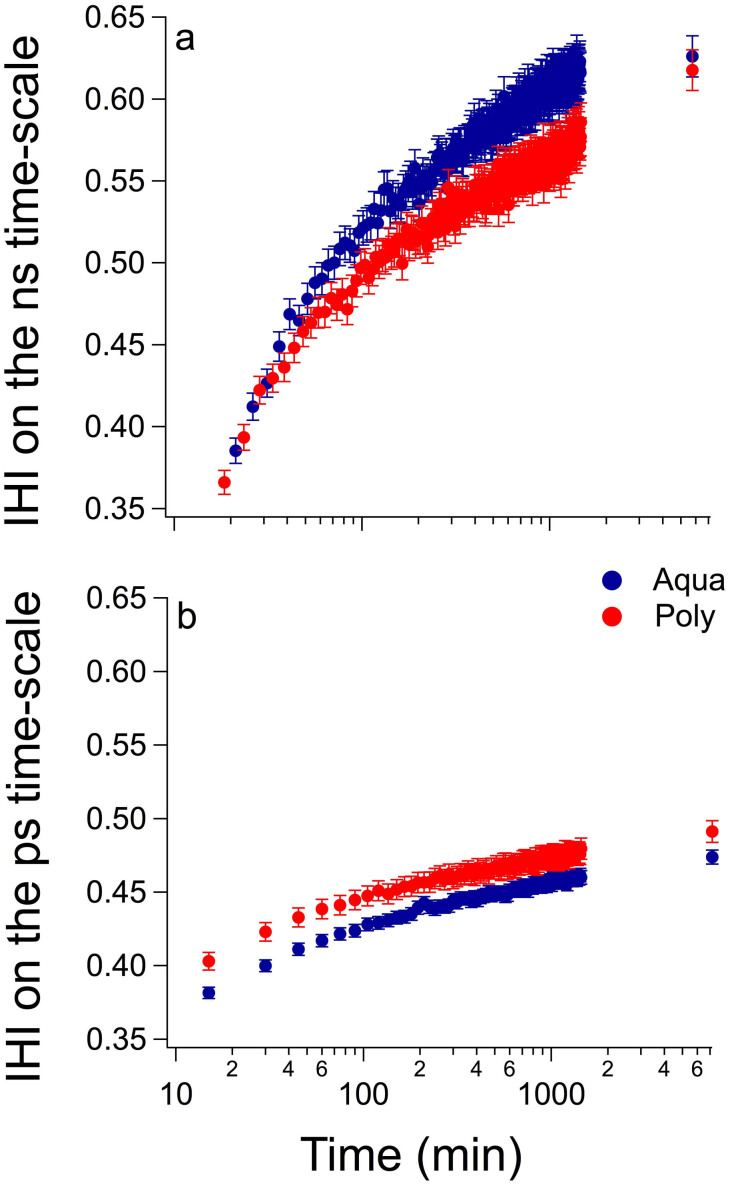
At the ns time-scale, the immobile protons are bound faster to the microstructure of the Aqua cement, although the total amount of immobile protons is similar for both cements after 5 days (a). At the ps time-scale, more protons are bound to the microstructure of the Poly cement, but a similar pattern is observed for both cements (b). In both curves the immobile hydrogen index (IHI) is plotted as a function of time in a log scale.

At the ns time-scale, the mobility of the slower and longer polyacid chains in the materials' structure was assessed using the high-resolution backscattering spectrometer IN10. From this data we observed that the setting is faster for the less viscous cement (Aqua) during the first 24 h of hydration. Interestingly, the IHI index is almost the same at the 5^th^ day (Figure 3a). This agrees with previous work, which identified the crosslinking process during setting to be mainly completed in one day^29^. To assess the mobility of the faster and smaller water molecules in the materials' structure at the ps time-scale, the backscattering spectrometer IRIS was used. Our data revealed that a larger amount of water molecules is incorporated into the structure of the more viscous material (Poly) already after 10 min (Figure 3b). After 5 days, the IHI remains higher for the Poly at the ps time-scale, but this process has not yet reached a plateau for either of the cements. This corroborates previous work, which demonstrates that the hydration process lasts several months^20^,^45^.

**Figure 4:**
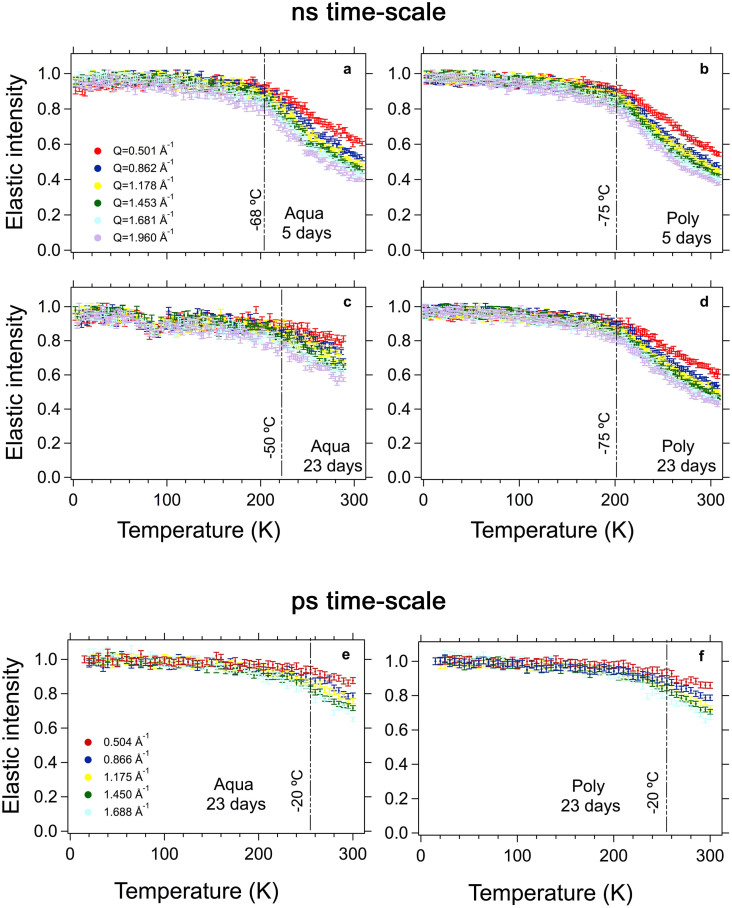
An increased amount of larger amplitude motions was detected at 205 K (−68°C) for the Aqua (a) and at 200 K (−75°C) for the Poly (b) at the ns time-scale. Furthermore, the observable drop in intensity at the lowest value of Q suggests differences in the diffusivity of the liquid in both materials. For the 23 day-old samples, while no changes in the temperature of the activated motions could be detected for the Poly (d), a significant reduction of the amount of mobile protons for the Aqua (c) is noticed, implying that important changes occurred in the Aqua structure. At the ps time-scale, a similar degree of proton mobility was observed for both materials after 23 days (e, f).

Proton mobility was also assessed as a function of temperature by following the elastic signal of the GIC from 2 K (liquid Helium temperature), when all motions are frozen, until body temperature (Figure 4, see supplementary information online for details). As expected, the intensity of the elastic line decreases with increasing temperature. However, the decrease of the elastic intensity deviates from linearity starting at the temperatures marked by the vertical lines in Figure 4. This phenomenon is related to the onset of non-harmonic motions, such as proton diffusion or rotation. At the ns time-scale, although no real difference (within the experimental error) in the number of mobile protons could be identified after 5 days from the start of the mixture, it was possible to differentiate the activation temperature for the protons in each sample (Figures 4a and 4b). The difference in the activation temperatures observed for the Poly and Aqua samples can be related to differences in their pore structure^35^,^50^. While no effect of ageing on proton mobility could be detected for the Poly (Figure 4d), ageing reduces proton mobility for the Aqua (Figure 4c) at the ns time-scale. At the same time, an increase in the temperature necessary to activate the motions is observed here for the Aqua. The increase in the activation temperature means that less energy is necessary to produce proton mobility, thus suggesting that either the probed motions are faster than the time window of the instrument or that a different proton population is present with ageing. In order to confirm which of these phenomena happens with ageing, we analysed the spectra applying the EFW method at the ps time-scale. In the aged samples, no difference in the amount of immobile protons was observed for the faster time-scale (Figures 4e, 4f). This indicates that the proton dynamics is very similar in both materials at the ps time-scale. The drastic changes observed in Figure 4c thus suggest that the longer polyacid chains are continuously being incorporated in the porous structure, and most likely the levelling of the elastic intensity is related to a new proton population present in the smaller pores.

### The flexural strength of the dental cements differed with ageing

In order to observe the evolution of the GIC strength as a function of maturation time, biaxial flexural strength (BFS) tests were performed after storage of the samples for 24 h, 7 and 32 days. This test, described in details by Dowling and co-workers, has been more recently proposed as an alternative method to the compressive strength test for encapsulated GIC^51^. Significant differences in biaxial flexural strength for the investigated materials within each period were identified by one-way analysis of variance (p < 0.001), followed by Tukey HSD post hoc test. Despite the expected increase in the mechanical strength due to post-hardening with ageing^45^,^46^, for Aqua the measured strength was constant throughout the maturation period (see Table 1).

## Discussion

Based on X-ray imaging, we show that the poor adaptation of the restorative materials within the dental cavity walls (Figures 1e, 1f ) and the network of pores and cracks (Figures 1a, 1c, 1e) in the GIC are worthy of concern. Furthermore, we found that the initial micro-porosity within GIC (threshold interval for 5-day-old samples: Aqua 2.9–4.5 vol%, Poly 2.0–2.9 vol%) was smaller than the porosity levels previously reported in microscopic assessments of these cements, ranging between 6–9%^38^,^39^. The smaller total porosity observed for the Poly cement can mainly be explained by the mechanical mixing of this material, which is associated to a lower probability of air inclusions^41^,^52^. It should be emphasized that these optimistic porosity results reflect the limitation of the instrument to detect pores smaller than 7 μm. For instance, a previous study using μCT found negligible porosity in a restorative GIC when using an instrument with coarser resolution^41^. Additionally, the existence of nano-pores, ranging between 2 and 400 nm, was confirmed in the investigated GIC by nitrogen adsorption experiments (see details of these results in the supplementary information). Consequently, when taking the micro- and nano-pores into account, the total porosity of the investigated GIC falls into the range of previously reported data.

Additionally, a reduction of pore sizes in the microstructure (threshold interval for aged samples: Aqua 1.9–3.8 vol%, Poly 1.4–2.6 vol%, Figures 2c, 2d) as well as in the nanostructure (see Table S1 in the supplementary information for nitrogen adsorption results) was observed with cement maturation. This result corroborates the reported increase in mechanical strength of GIC with time^44^,^45^,^46^. In this study, however, an increase in flexural strength with ageing was identified only for Poly. For Aqua, the flexural strength remained unchanged throughout the investigated period (see Table 1). Although the mechanical strength of GIC is largely influenced by the presence of surface flaws^53^, the total porosity^41^,^42^,^43^, the mixing technique^40^,^54^,^55^,^56^, the powder to liquid ratio^57^, the molar mass and concentration of the polyacid in the GIC^58^,^59^, our results indicate that the development of the micro- and nanostructure also plays an important role. As shown here, a less significant change both in the microstructure and the nanostructure (see Table S1 in the supplementary information) is observed for Aqua.

Other than the porous structure, cracks were identified in GIC restorations aged in water at body temperature (Figure 1a), despite the surface protection with a resin layer (indicated to minimize desiccation and water uptake) and the absence of mechanical loading. The presence of cracks in GIC has traditionally been attributed to the dehydration of the cement during preparation for SEM analysis^38^,^43^. The results from this study, on the contrary, suggest that the cracks are inherent to the investigated GIC (Figure 1a), possibly as a result of proton mobility within the materials' microstructure (Figure 4). Crack propagation due to the mobility of the liquid can, together with the low flexural strength of the material^12^,^13^, help to explain the limited durability of GIC restorations^1^,^4^. These cracks are not visible in conventional dental radiographies (see Figure S1 in the supplementary information online for a comparison of conventional images with those acquired with μCT).

Regarding proton mobility within GIC, neutron imaging revealed the presence of liquid within the materials' microstructure (Figures 1b, 1d, 1f). The mobility of the liquid within cracks and pores^36^ has been connected to the materials' longevity in construction cements. This occurs because the pressure exerted by water adsorbed on the walls of a crack favours the cracking process, with consequent reduction in the cements' strength^60^. Furthermore, as the hydration process of construction cements evolves with time, progressively more hydrogen becomes bound to the materials' structure^13^,^17^,^35^,^45^, resulting in an increase in strength^45^,^46^. Consequently, regardless the differences between dental and construction cements, the knowledge acquired previously from construction cements using QENS was applied here, since GIC are also water-based cements. The incorporation of hydrogen within the cement structure during setting was confirmed for the investigated GIC (Figure 3). The IHI observed at the ns time-scale (i.e. probing mostly the mobility of the polyacid molecules) was larger for the Aqua cement during the first 24 h, but was similar for both cements after 5 days (Figure 3a). At the ps time-scale (i.e. probing mostly the mobility of the water molecules), however, the IHI for the Poly is higher than the Aqua during the whole investigated hydration period (Figure 3b). Unbound liquid is still found on the 5^th^ day after preparation of both GIC (Figures 4a, 4b). Despite the similar degree of proton mobility assessed in the ps time-scale after 23 days (Figure 4e, 4f ), an intermediary proton population was detected in the Aqua cement in the ns time-scale (Figure 4c), which can further explain its lower strength. Differences in proton mobility between the investigated GIC were expected due to their distinct formulation and powder to liquid ratio. Nonetheless, the considerable proton mobility observed in both GIC cements may shed light on the limited longevity of these dental restorative materials. Future research aiming to decrease liquid mobility may thus help to improve GIC.

## Methods

### Preparation of the cement pastes

Both investigated GIC cements consisted of a powder containing irregular particles of fluoroaluminosilicate glass that undergo an acid-base reaction on contact with the liquid. In the Poly cement (Ionofil Molar AC, Voco GmbH, Germany), the liquid contains a polyacid solution, while in the Aqua (Aqua Ionofil Plus, Voco GmbH, Germany), as the polyacid is freeze-dried and incorporated into the powder, the liquid is water. The cement pastes were mixed according to the manufacturer's recommendation. For all experiments, the Aqua samples (Aqua Ionofil Plus, Lot 1149136) were mixed by hand using a powder to liquid ratio of 5.6:1 by weight. For the more viscous GIC, Poly, the material was either encapsulated (Ionofil Molar AC, Lot 1141120) and mixed in a mechanical agitator (CapMix Capsule Mixing Device, 3M ESPE AG, Germany) at 4650 rpm for 10 s, or hand-mixed when using its hand-mixed equivalent (Ionofil Molar, powder Lot 1143477, liquid Lot 1144042), in a powder to liquid ratio of 3.7:1. The Poly hand-mixed equivalent was adopted only for the measurements using the IRIS spectrometer, due to difficulties in the experimental set-up. For the hand-mixed GIC, the appropriate weight of powder and liquid were dispensed, and the powder was divided into two equal portions. The cement paste was thoroughly mixed using a plastic spatula on paper blocks within 60 s, until a visibly homogeneous mass was obtained. Preparation of the cement pastes was carried out at room temperature (20 ± 1°C) and controlled humidity (50 ± 5%). The samples were aged in water at 37°C (body temperature), except for the nitrogen adsorption experiments (see supplementary information for details).

### Biaxial flexural strength

Six disc-shaped specimens (10.0 ± 0.1 mm diameter, 1.0 ± 0.1 mm height) were prepared from GIC for the biaxial flexural strength test. The cements were inserted in cylindrical moulds and pressed against polyester strips. The specimens were then secured with flat metallic clamps and stored in deionized water at 37°C for 24 h, 7 days or 32 days. After storage, the specimens were placed on a cylindrical ring support (3.8 mm radius) over a thin sheet of rubber for a uniform distribution of stresses under loading. The specimens were loaded centrally with a ball-shaped indenter in a universal testing machine with crosshead speed of 1 mm/min, and the load at fracture (P) was recorded. The height of the specimens at the site of fracture (h) was measured with a micrometer. The biaxial flexural strength (BFS) was calculated using equation (1).
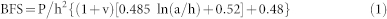
where a is the radius of the support, v is Poisson's ratio (0.3 for glass ionomer restoratives^61^), h is the height of the specimen and P is the measured load at fracture.

### X-ray and neutron imaging

The first set of experiments includes imaging obtained from X-ray and neutron tomography of various teeth restored with GIC at distinct periods of time after preparation of the mixture, thus providing valuable information about the liquid content and its evolution within the microstructure of the dental cements. Extracted human premolars were prepared and restored with GIC. X-ray tomography was performed on aged samples: initially 5 days after preparation of the cement mixtures and later after 79 days for the Aqua and 93 days for the Poly. The restored teeth were stored in deionized water at 37°C during aging.

The X-ray images were obtained using a μCT operated at 100 kV and 100 μA in order to obtain good contrast. For every tomography, 1000 projections on 360° were acquired. Three images were recorded for each projection with an exposure time of 1.5 s for filtering, processing and averaging in order to correct for background artefacts. The flat panel detector pixel size was 50 μm and the spatial image resolution was 6.9 μm due to the magnification geometry obtained using a cone beam.

The neutron images were obtained using the instrument V7 (CONRAD-2) at Helmholtz Zentrum Berlin (HZB Berlin, Germany). A neutron flux density of 10^7^ per cm^2^/s at a beam collimation (L/D, where L is the pinhole to sample distance and D is the diameter of the pinhole) of 350 reached the sample. For each tomography 600 projections on a range of 360° were obtained. Three images were recorded and median filtered for each projection on a CCD detector of 6.4 μm pixel size. The similar pixel size of the X-ray and the neutron instruments allowed assessing pores larger than 7 μm.

All images acquired using X-rays and neutrons were reconstructed using the software Octopus (inCT, Belgium) and analysed with the software VG Studio Max (Volume Graphics GmbH, Germany). Additionally, pore analysis from the X-ray tomography data was performed using the software Avizo (FEI Visualization Sciences Group, United States). The resolution of the X-ray μCT allowed assessing pores larger than 7 μm.

### Quasi-elastic neutron scattering

A second set of experiments investigated proton mobility within the dental cements by means of quasi-elastic neutron scattering (QENS), which was then analysed using the area under the spectra within a determined interval, known as the elastic fixed window (EFW) method. The evolution of the elastic intensity as a function of temperature allows probing the mobility of the protons within the structure. The hydrogen mobility in the aged GIC samples (5- and 23-day-old) was determined at the ns time-scale and after 23 days at the ps time-scale. Moreover, by following the ratio between the elastic intensity that evolves upon the setting of the GIC (at each time point) by the total intensity over time, it was possible to follow the hydration process of the GIC up to 5 days at both ns and ps time-scales. This ratio determines the immobile hydrogen index, IHI.

The evolution of the elastic intensity was assessed at the nanosecond (ns) time-scale using the IN10 spectrometer at Institut Laue-Langevin (Grenoble, France) and at the pico-second (ps) time-scale using the IRIS backscattering spectrometer at the ISIS Facility (Didcot, United Kingdom). At the ns time-scale, the mobility of the slower hydrogen protons present in the long polyacid chains could be probed. Additionally, the mobility of the fast hydrogen protons, such as those observed in water molecules, could be probed at the ps time-scale.

For the QENS measurements, the GIC samples were matured at 37°C up to 28 days. The samples were wrapped in aluminium foil and then mounted inside an aluminium sample holder isolated with indium wire. Neutron scattering measurements were initiated 14–18 min after the preparation of the mixture. For the IN10 spectrometer, an elastic energy resolution, *ΔE*, of 1 μeV at full-width at half maximum (FWHM), corresponding to 4 ns, was achieved by neutrons scattered with λ = 6.271 Å. This energy was constant within an angular range of 11° < θ < 155°. For the IRIS spectrometer, an elastic energy resolution of 17.5 μeV at FWHM, corresponding to an upper experimental observation time of ~100 ps, was achieved by neutrons scattered with λ = 6.7 Å. This resolution was constant over the entire angular scattering range of 25° < θ < 160°. In both spectrometers the angle between the plane of the sample and the incident neutron beam was 135° and the Q, scattering vector, range varied from 0.501 to 1.960 Å^−1^. Therefore, data from the last detectors were self-shielded by the edge of the sample holder and thus discarded.

### Additional details

Details from X-ray and neutron imaging techniques can be found in the supplementary information online. Neutron scattering experiments, data reduction and data analysis are also provided in the supplementary information, as well as the nitrogen adsorption experiments.

## Author Contributions

A.R.B. and H.N.B. conceived the experiments, performed the data analysis and wrote the manuscript. A.R.B., J.J., B.L., N.C.R.M., M.T.F.T. and H.N.B. performed the experiments and data reduction. D.V.O., M.T.F.T., N.K., M.S. and T.S. provided expertise with instrumentation and gave insight on the data analysis. IM gave support for the imaging experiments. This manuscript was approved by all co-authors in its current version.

## Supplementary Material

Supplementary Information Supplementary information

## Figures and Tables

**Table 1:** Mean biaxial flexural strength (MPa) and standard deviation for the two commercial glass ionomer cements. Similar letters do not indicate significant differences (Tukey HSD post hoc test, p < 0.05)

	Biaxial flexural strength (MPa)		
Material	24 h	7 days	32 days
Poly (VOCO Ionofil Molar AC)	66 ± 6^bc^	76 ± 7^ab^	84 ± 13^a^
Aqua (Aqua Ionofil Plus)	54 ± 4^cd^	52 ± 5^cd^	49 ± 9^d^
