# Supplementary material for: How mobile are protons in the structure of dental glass ionomer cements?
Source: Sci Rep. 2015 Mar 10;5:8972. doi: 10.1038/srep08972 (PMC4354011; doi:10.1038/srep08972)
Supplement: Supplementary Information [file srep08972-s1.pdf]

Supporting Information

How mobile are protons in the structure of dental glass ionomer cements?

Ana R. Benetti[1], Johan Jacobsen[2,3], Benedict Lehnhoff[2], Niels C. R. Momsen[2], Denis V. Okhrimenko[4], Mark T. F. Telling[5,6], Nikolay Kardjilov[7], Markus Strobl[3], Tilo Seydel[8], Ingo Manke[7], Heloisa N. Bordallo[2,3*]

[1]Department of Odontology, Faculty of Health and Medical Sciences, University of Copenhagen, DK-2200, Copenhagen, Denmark; [2]The Niels Bohr Institute, University of Copenhagen, DK-2100, Copenhagen, Denmark; [3]European Spallation Source ESS AB, PO Box 176, SE-221 00 Lund, Sweden; [4]Nano-Science Center, Department of Chemistry, University of Copenhagen, DK-2100, Copenhagen, Denmark; [5]ISIS Facility, Rutherford Appleton Laboratory, Chilton, Oxon, UK OX11 0QX; [6]Department of Materials, University of Oxford, Parks Road, Oxford, UK; [7]Helmholtz Zentrum Berlin, D-14109, Berlin, Germany; [8]Institut Laue-Langevin, BP 156, F-38042, Grenoble, France.

*Correspondence and requests for materials should be addressed to: H.N.B (bordallo@nbi.ku.dk)

Details about the restorative procedure. Class I cavities (depth 2 mm, width 2 mm) were prepared on the occlusal surface of extracted human upper premolars, using a pear-shaped diamond rotary instrument. The teeth were kept in water until immediately before the restorative procedure. The cavities were dried with absorbent paper without excessive desiccation. Subsequently, the glass ionomers were prepared according to the procedure described in the main text and inserted into each cavity. The material was accommodated at the bottom of the cavity and against the cavity margins using a metallic instrument. Before the cements were completely set, a layer of resin (Easy Glaze, Lot 1203261, Voco) was immediately applied on the surface of the restorations. The resin was light activated for 30 seconds using a LED lamp (bluephase, Ivoclar Vivadent, Liechtenstein) at 950 mW/cm^2. The application of the resin on the surface of the restorations is indicated to minimize desiccation and water uptake from the GIC immediately after placement. The teeth were subsequently stored in water at 37°C for 5 days prior to the first imaging experiments. After obtaining the images, the teeth were again stored in water (Aqua 79 days, Poly 93 days) until images of the aged samples were acquired. Conventional images of the extracted teeth were also acquired with a dental X-ray equipment for comparison of the resolution of the images obtained in the daily practice of a dentist (Figure S1).

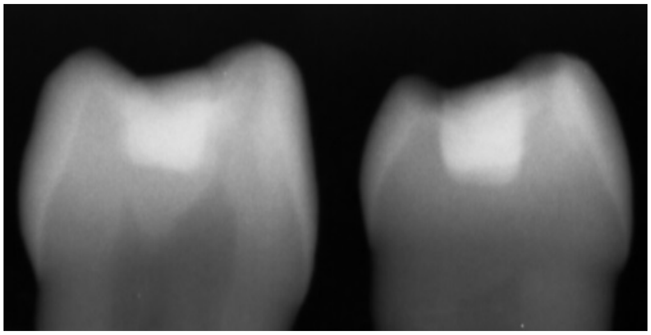

Figure S1. In a conventional image using a dental X-ray, the porosity and cracks within the restorations are not visible due to the limited resolution and short exposure period. Both teeth were restored with GIC (Aqua: left; Poly: right).

X-ray imaging. The restored teeth were mounted in a sample holder and remained immersed in water during acquisition of the X-ray images, to avoid desiccation. The x-ray images were obtained using a micro-focus X-ray tube with a 2048 x 2048 pixels amorphous-Si flat panel detector, every pixel having a pixel size of 50 x 50 µm. In order to give best possible magnification, while keeping a high photon flux, a compromise was found for the source-object distance, thus reducing the effective pixel size to 7 µm. The source to detector distance was 500 mm and the source to object distance was 70 mm. For every tomography, 1000 projections on 360° were acquired. Ten flat-field and 10 dark-field images were taken in the beginning and the end of each measurement for post-processing image normalization. A cone beam algorithm was used for reconstruction of the X-ray images.

Neutron imaging. The neutron images were obtained using the neutron instrument V7 (CONRAD-2) located at the Helmholtz Zentrum Berlin (HZB Berlin, Germany). Here, neutrons are produced in a reactor and a neutron flux density of 10^7 per cm^2/s at a beam collimation (L/D, where L is the pinhole to sample distance and D is the diameter of the pinhole) of 350 reached the sample. To avoid desiccation during image acquisition, the restored teeth were mounted in a sample holder in which the roots were surrounded by a wet sponge. For every tomography, 600 projections on a range of 360° were obtained. Three images were recorded and median filtered for each projection. In total, 1800 images were obtained from each sample on a CCD detector of 6.4 µm pixel size. During the measurements, a high-resolution set-up was applied, thus allowing the best possible neutron resolution up-to-date. Ten flat-field and 10 dark-field images were taken in the beginning and the end of each measurement. Neutron images were reconstructed using a parallel beam reconstruction based on a filtered back projection algorithm. Conventional filtering was applied under reconstruction to allow better image quality.

Gas adsorption measurements (Surface area and Porosity determination). This technique utilizes gas adsorption in order to collect information about surface area and pore volume.[1] It is one of the most widely used methods for pore structure characterization,[2] measuring pore diameters up to 400nm. However, gas adsorption studies only measure pores that have contact to the surface of the samples as opposed to X-ray and neutron imaging that investigates the whole material volume.

In order to get insight about micro (<2 nm), meso (2-50 nm) and macropore (50-400 nm) evolution during the hydration process in the GIC, gas (nitrogen) adsorption measurements were performed at liquid nitrogen temperature (-196°C) using a Quantachrome Nova 2000e Surface Area Analyzer. The specific surface areas were determined from nitrogen adsorption isotherms in the relative pressure range $0.1 < P/P_0 < 0.3$, using the BET equation.[3]

Four cylinders (diameter 3 ± 0.1 mm, height 6 ± 0.1 mm) from each GIC were fabricated. The GIC were prepared following the procedure described in the main text of the manuscript and inserted in cylindrical moulds. The specimens were then covered with polyester strips and

---

[1] Yania, A.J. & Hansen, W. Pore structure of hydrated cement determined by mercury porosimetry and nitrogen sorption techniques. *Mat. Res. Soc. Symp. Proc.* **137**, 105-119 (1969).

[2] Lowell, S., Shields, J.E., Thomas, M.A. & Thommes, M. Characterization of porous solids and powders: surface area, pore size and density. Kluwer Academic Publishers, Netherlands (2004).

[3] Brunauer, S., Emmett, P.H. & Teller, E. Adsorption of gases in multimolecular layers. *J Amer Chem Soc* **60**,309-319 (1938).

clamped between metallic cylinders. Subsequently, the specimens were protected with Parafilm M (Bemis Company Inc, Neenah WI, USA) and stored dry at 37°C during 30 min, 24±1 h, 7±1 days or 30±2 days. Prior to the measurements, the samples (in average 1.3 g) were degassed by heating to 150 °C in vacuum (<10^{-3} torr) for 24 h. This procedure ensured that all pre-adsorbed water from the surface was removed. For all samples, the BET plots were linear in the relative pressure range examined, confirming the applicability of the BET equation. The pore sizes were estimated using t-plots for micro-pores in the pressure range $0.4 < P/P_0 < 0.9$. These t-plots were linearized in the pressure rage $0.4 < P/P_0 < 0.8$ and the micro-pore volume and surface area were estimated using the de Boer equation.[4] Total pore volume was estimated at P/P_0 equal to 0.98 from the total amount of adsorbed nitrogen.

A typical isotherm from the adsorption investigations can be seen in Figure S2. Here the isotherms from a nitrogen adsorption measurement at -196°C for the Poly GIC are visualized during different stages of setting. These isotherms appear to be close to type I and type II isotherms, which represent materials that contain primarily micro-pores or primarily meso- and macro-pores, respectively. Thus, we can conclude that the samples have a large distribution of pores that ranges from micro- to meso- and macro-pores. However, as there appears to be a change in the slope during filling at relative pressures $P/P_0 < 0.1$ than for $P/P_0 = [0.80 - 0.98]$, it is most likely that this material contains a larger volume of micro-pores, i.e pores with a diameter smaller than 2nm. The BET determined surface areas as well as the pore volume distribution over time for the two GIC are given in Table S1.

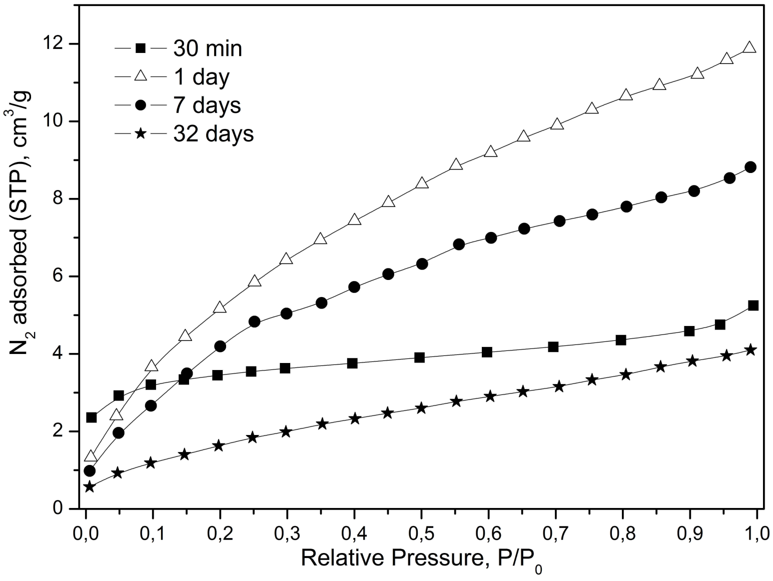

Figure S2. Nitrogen adsorption isotherms at -196°C for the Poly sample stored for 30 min, 24 h, 7 days and 32 days.

[4] de Boer, J.H., Lippens, B.C., Linsen, B.G., Broekhoff, J.C.P., van den Heuvel, A., Osinga, J.T. The t-curve of multimolecular N_2-adsorption. *J Colloid Interface Sci* **21**, 405–414 (1966).

Table S1. BET determined surface areas as well as the pore volume distribution over time for the Aqua and Poly samples. The total pore volume is also given in vol%.

	Setting time	Poly		Aqua	
Surface area (m^2/g)	30 min	12.6		9.1	
	24 h	22.5		20.6	
	7 days	20.7		18.4	
	32 days	6.8		12.2	
Total pore volume (10^{-3} cm^3/g) Pores from <400 nm	30 min	8.1	2.0 vol%	6.9	1.3 vol%
	24 h	18.0	3.5 vol%	14.6	2.7 vol%
	7 days	14.0	2.7 vol%	12.2	2.3 vol%
	32 days	6.3	1.1 vol%	9.6	2.2 vol%
Micropore volume (10^{-3} cm^3/g) Pores < 2 nm	30 min	5.0		4.5	
	24 h	13.0		10.4	
	7 days	8.6		8.4	
	32 days	2.0		5.1	
Meso/macropore volume (10^{-3} cm^3/g) Pores from 2 - 400 nm	30 min	3.1		2.4	
	24 h	5.0		4.2	
	7 days	5.4		3.8	
	32 days	4.3		4.5	

Interestingly, the total volume of pores obtained from the gas adsorption measurements is twice as large in the Aqua samples. Moreover, the total micro-, meso- and macro-pore volume increases for both samples during the first 24 h of maturation, but decreases considerably for the Poly sample as time evolves. These results, however, must be considered with caution since drying of the samples might cause pore-coarsening and structural changes on the nano-level.[5] For instance, the specific surface area of Portland cement obtained from water vapour adsorption is normally greater than that calculated from nitrogen adsorption.[5] Moreover, as previous reported, correlations between compressive strength of samples and their corresponding specific pore characteristic values might not show any significant relationships.[6] This implies that information on the specific pore structure alone is not sufficient to draw conclusions about the strength of the dental cements and that water dynamics also plays an important role on their mechanical properties.

Hydration and temperature evolution of the elastic line. GIC cement pastes were prepared, as described previously in the main text, placed on an aluminium foil envelope, and flattened out using a steel rolling pin. Subsequently, the sample was enclosed in an aluminium sample holder, sealed with indium wire and mounted either inside the high resolution neutron backscattering instrument IN10 (located at the ILL, France) or at the neutron backscattering spectrometer IRIS (located at ISIS, UK). The mass of the sample holder and of the aluminium foil envelopes, with and without the samples, was registered before and after the final measurement to ensure that no liquid was lost throughout the experiment. The IN10 data was reduced using the software

[5] Aono, Y., Matsushita, F., Shibata, S. & Hama, Y. Nano-structural changes of C-S-H in hardened cement paste during drying at 50°C. *J Adv Concrete Technol* **5,** 313-323 (2007).
[6] Azizi, N. A gas adsorption porosimetry of specific pore characteristics of Portland cement prepared by two placement methods. University of California, San Francisco, ProQuest, UMI Dissertations Publishing, 1465478 (2009).

package Lamp (ILL, Grenoble, France), while the IRIS data was analysed using DAVE. A vanadium sample was measured to normalize for the detector efficiency, and the signal of an empty sample holder was obtained to evaluate the instrument background. In all experiments, the samples were mounted so the angle between the sample and the incident neutron beam was 135°, which resulted in shading of the higher angle detectors during data collection.

In order to follow the hydration process during the first 24 h, the elastic signal was recorded every 5 minutes during the IN10 data collection. For the experiment using IRIS, spectra were collected every 15 minutes (total accumulated ISIS proton current 40 μamps per measurement) over a 24 h period. Data collection was initiated at body temperature (310K, 37°C) approximately 15 minutes after the start of the mixture. After 24 hours, the samples were removed from the instrument to mature in an oven at 37°C up to 5 days, and the evolution of the elastic line as a function of temperature was collected between 2 and 310 K. To analyse the evolution of the elastic line as a function of temperature of the aged GIC, two additional sets of samples were prepared and mounted inside the sample holders, following the same procedure described above. These samples were aged at body temperature for 23 days prior to the experiments.

Evaluation of the immobile hydrogen index (IHI). The IHI, defined as the ratio between the elastic intensity that evolves with setting of the GIC (at each time point) by the total intensity registered by the spectrometer, which is constant over time, was obtained. For the IRIS experiment, to address the hydration process on the ps time scale, the integrated elastic intensity, determined by the width of the resolution function of the instrument, ΔE 17.5 μeV, was measured as a function of time and divided by the total elastic intensity of the samples determined from the 5-day-old samples. During the IN10 experiments, the hydration process was characterized using the elastic fixed window method (Doppler off), while the total signal at 5 days was obtained using the inelastic configuration of the instrument (Doppler on). As a consequence the flux on the sample is different and flux normalization was also required.

The main difference between the hydration measurements registered at IRIS and IN10 stems from the manner in which the instruments function. On IRIS, the elastic and quasi-elastic signals are measured at the same time. However, on IN10 one chooses whether to measure the elastic or the quasi-elastic signal. The elastic signal is measured with the Doppler drive turned off while the quasi-elastic signal requires the Doppler drive to be turned on. On IN10 the hydration experiments were carried out with the Doppler turned off during the first 24 h of setting of the cements. This allowed maximal flux to register the elastic intensity. The measurement of the matured samples after 4 days, however, was carried out both with the Doppler drive turned on to measure the quasi-elastic signal, and off to measure the elastic signal.

Evaluation of the temperature-dependence of dynamical relaxation processes. Measurements of elastic intensity of the 5-day-old samples as a function of temperature from 2 to 310 K (see Figure 4) allow identifying when proton movements are first activated with increasing of temperature. It is expected that if a dynamical transition takes place, the temperature-dependence of the dynamical relaxation processes will cause a change in the slope of the observed elastic intensity.
